# Evaluation of muscle volume and degeneration after total hip arthroplasty: a comparison of the posterolateral approach and the anterolateral supine approach

**DOI:** 10.1186/s13018-021-02291-y

**Published:** 2021-02-18

**Authors:** Taku Ukai, Goro Ebihara, Haruka Omura, Masahiko Watanabe

**Affiliations:** Department of Orthopedic Surgery, Surgical Science, Tokai University School of Medicine, 143 Shimokasuya, Isehara, Kanagawa 259 1193 Japan

**Keywords:** Total hip arthroplasty; Posterolateral approach; Anterolateral approach; Muscle volume; Muscle degeneration

## Abstract

**Background:**

Muscle strength around the hip after total hip arthroplasty (THA) is crucial for preventing dislocation and limping. This study aimed to assess and compare muscle volume and degeneration after THA using the posterolateral (PL) and anterolateral (AL) approaches.

**Methods:**

Sixty-four hips in 64 patients who underwent primary THA were retrospectively analyzed. Patients were segregated into the PL group (35 hips) and AL group (29 hips) for evaluating pre- and postoperative muscle volumes and degeneration around the hip. Computed tomography (CT) examinations were performed preoperatively and 6 months post THA. The muscle volume and Hounsfield units (HU) of the gluteus maximus (G-max), gluteus medius (G-med), tensor fasciae latae, internal obturator muscle, and external obturator muscle were measured.

**Results:**

In the PL group, the postoperative muscle volume of the G-max significantly increased than the preoperative muscle volume. In contrast, the postoperative muscle volume of the internal obturator muscle was significantly lower than the preoperative muscle volume. The postoperative HU of the internal and external obturator muscles were significantly lower than the preoperative HU. In the AL group, the postoperative muscle volumes of the G-max, G-med, and tensor fasciae latae significantly increased than their preoperative muscle volumes. The postoperative HU of the G-med and tensor fasciae latae were significantly higher than the preoperative HU values.

**Conclusion:**

The PL approach can lead to degeneration of the internal and external obturator. The AL approach is more beneficial for recovering the G-med, tensor fasciae latae, and internal obturator muscle than the PL approach.

## Background

Total hip arthroplasty (THA) is performed worldwide and is beneficial not only for relieving pain of the hip but also for recovering the hip function. Particularly, muscle strength and recovery after THA can affect gait, causing limping and Trendelenburg gait, which can contribute to patients’ dissatisfaction. Thus, evaluating muscle recovery after THA is essential. Computed tomography (CT) is commonly used for the quantification of muscle volume around the hip [1, 2], and some studies have reported the investigation of muscle volume after THA using CT [3–6].

Several surgical approaches have been reported for THA, such as posterolateral, direct lateral, direct anterior, and anterolateral supine approach. The posterolateral (PL) approach is the most basic approach, and the implantation of the stem component is easier in the PL approach than in other approaches [7, 8]. However, the PL approach entails resection of short external rotator muscles [9], which may affect the postoperative weakness of the external rotation of the hip. In contrast, the anterolateral (AL) approach does not need any muscle resection because it is an intermuscular approach between the tensor fasciae latae and gluteus medius (G-med). Thus, muscle recovery after THA may vary depending on the approach. However, there are no reports evaluating muscle volume and degeneration after THA using the PL and AL approaches. CT examinations can evaluate not only muscle volume but also degeneration. In addition, Hounsfield units (HU) are useful for evaluating degeneration because HU vary depending on densities: air, − 1000 HU; fat, − 100 HU; water, 0 HU; muscle, 30–50 HU; and bone, 400–1000. As muscle degeneration progresses, CT image intensity decreases [10].

In this study, we aimed to evaluate muscle volume and degeneration using CT for determining whether PL and AL approaches affect muscle volume and degeneration after THA.

## Materials and methods

### Population

For a moderate effect size (*d* = 0.5) determined using the Wilcoxon analysis (matched pairs) and a requirement of 80% power for detecting an existing difference, the required sample size was 28 patients. We retrospectively investigated patients who underwent THA between 2017 and 2020. All procedures were approved by the ethics committee at the authors’ institution (20R–021). Inclusion criteria included patients who underwent THA and pre- and postoperative CT scans. Exclusion criteria included patients who had prior ipsilateral hip surgery, spinal surgeries, or who are unable to walk by themselves before THA.

### Surgical procedure

The PL approach was performed in the decubitus position. After dissecting the short external rotators and removing the femoral heads, a cementless acetabular component (Pinnacle; DePuy Synthes, Leeds, UK) and cementless proximally porous-coated femoral component (S-ROM(A); DePuy Synthes, Warsaw, IN, USA) were placed. Femoral head sizes of 32 mm were used for all patients. After placement of the acetabular and femoral components, the short external rotators were recovered using suturing with the greater trochanter. The posterior capsule was also sutured. In all the patients, weight-bearing was allowed on the day after the surgery, as tolerated.

The ALS approach was performed in the supine position, and an image intensifier was used. A cementless acetabular component (Continium; Zimmer Biomet, Warsaw, IN, USA) and cementless proximally porous-coated femoral component (Fitmore; Zimmer Biomet, Winterthur, Switzerland) were placed. The vertical iliofemoral ligament, conjoint tendon, and anterior capsule were preserved. Similar to the PL approach, weight-bearing was tolerated on the day after the surgery.

### Radiographic evaluation

**Fig. 1:**
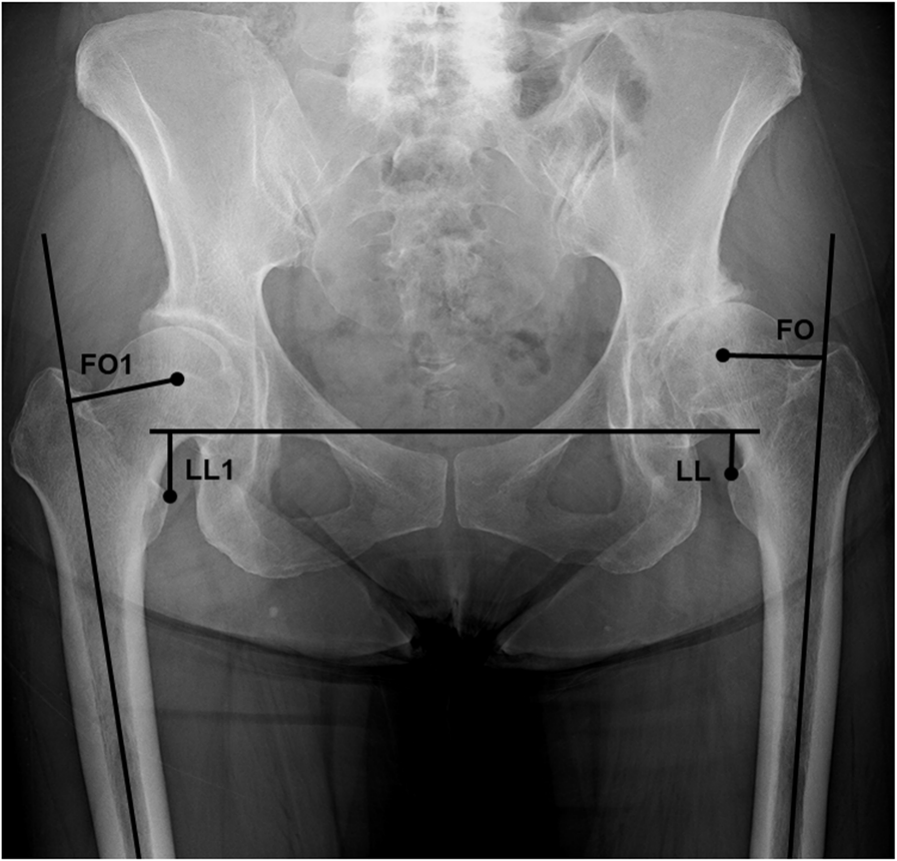
Measurement of leg discrepancy and femoral offset

The preoperative femoral offset was measured as the perpendicular distance between the center of the femoral head and the long axis of the femur. Preoperative leg length was measured as the perpendicular distance between the interteardrop line and the lesser trochanter. Leg length discrepancy was calculated as the affected side minus the healthy side (Fig. 1).

### CT evaluation

**Fig. 2:**
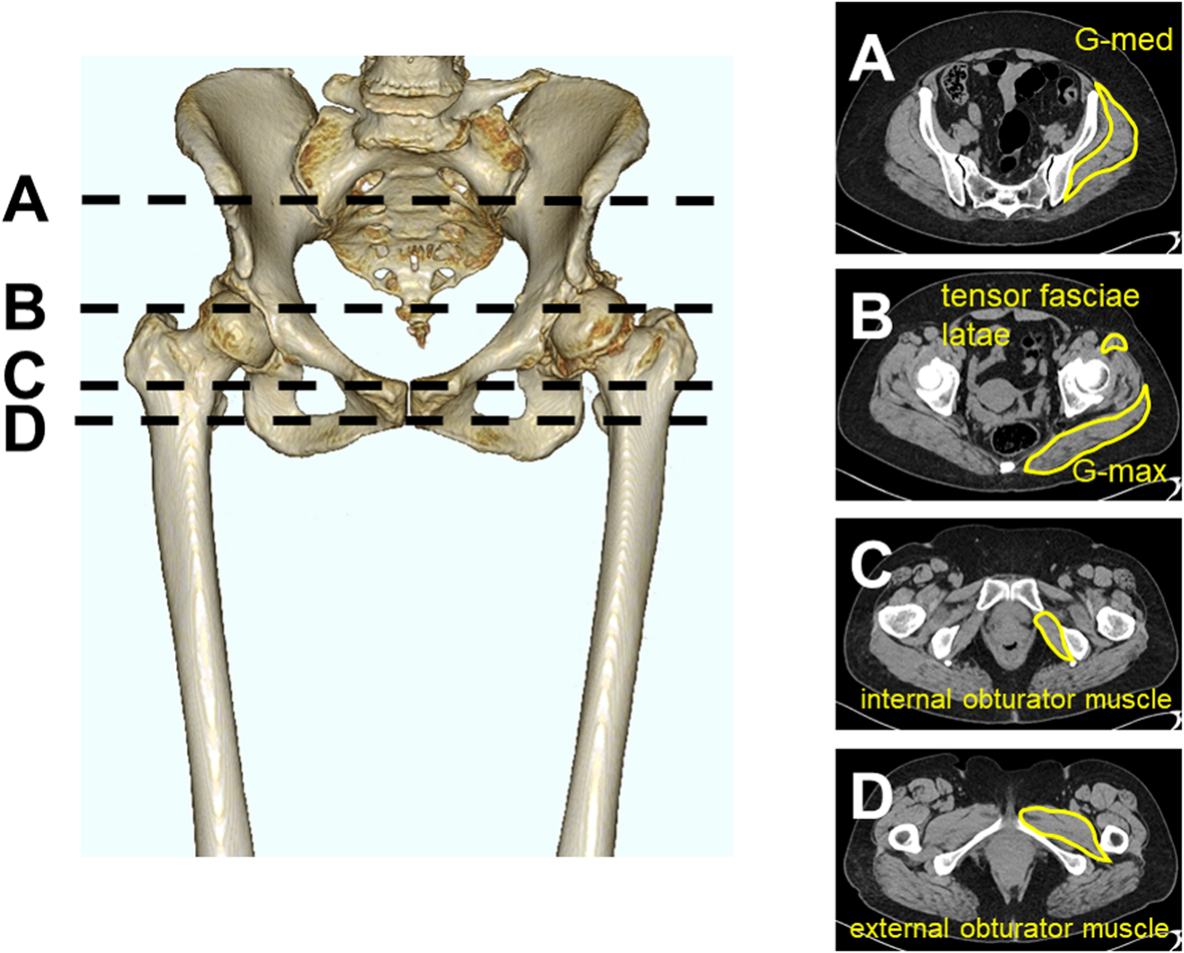
Cross-sectional analysis of muscle volume and Hounsfield units. **a** G-med was measured at the level of anterior superior iliac spine. **b** G-max and tensor fasciae latae were measured at the top of the greater trochanter. **c** Internal obturator muscle was measured at the level of the pubic tubercles. **d** External obturator muscle was measured at the level of the lesser trochanter

Pre- and postoperative CT images were acquired from the anterior superior iliac spine (ASIS) to the knee joint according to a standard protocol (Siemens, Germany; 120 kV, slice thickness 0.6 mm, 0.5–1 s scan time). Preoperative CT images were obtained a day before THA, and postoperative CT images were obtained 6 months after THA. The anterior pelvic plane (APP) was defined as the plane touching the most anterior points of the bilateral ASIS and pubic tubercles. The cross-sectional areas (CSAs) of the gluteus maximus (G-max), G-med, tensor fasciae latae, internal obturator muscle, and external obturator muscle were measured. The CSA of the G-med was measured on the plane perpendicular to the APP through the affected ASIS. The CSA of the G-max and tensor fasciae latae were measured perpendicular to the APP through the most proximal point of the affected greater trochanter. The CSA of the internal obturator was measured perpendicular to the APP through the pubic tubercles. The CSA of the external obturator was measured perpendicular to the APP through the affected lesser trochanters. The measured muscle volume was normalized for each patient’s body weight (mm^2^/kg). The border line of the muscle and other tissues was traced manually, and the muscle volume and HU were measured using the imaging analysis software DICOM in a 512 × 512 pixel format (Fig. 2).

### Statistical analyses

Statistical analyses were conducted using the SPSS statistical software version 26 (IBM Corp., Japan, Tokyo). Patients’ demographics and baseline characteristics were described and compared between the groups according to their distributions using Student’s *t*-test and chi-squared test. The Wilcoxon signed-rank test was used for evaluating pre- and postoperative muscle volumes and HU values of each group. Intraobserver and interobserver reliabilities were tested using intraclass correlation coefficients (ICCs), and their 95% confidence intervals (CI) were used for assessing the reliability of CSA measurement for each muscle [5, 11]. Two evaluators evaluated each patient with two replicates. All tests were performed at a significance level of *P* < 0.05.

## Results

The PL approach was performed on 35 patients (PL group; nine joints in men and 26 joints in women) with a mean age of 67.1 ± 9.9 years and a mean body mass index (BMI) of 26.5 ± 5.2 kg/m^2^. The mean preoperative leg discrepancy in the PL group was − 9.9 ± 9.1 mm. The mean preoperative offset in the PL group was 46.9 ± 7.4 mm. The AL approach was performed on 29 patients (AL group; three joints in men and 26 joints in women) with a mean age of 67.4 ± 10 years and a mean BMI of 22.6 ± 4.7 kg/m^2^. The mean preoperative leg discrepancy in the AL group was − 6.5 ± 7.2 mm. The mean preoperative offset in the AL group was 48.7 ± 8.7 mm. The preoperative BMI in the PL group was significantly higher than that in the AL group. The preoperative leg discrepancy in the PL group was significantly shorter than that in the AL group (*P* < 0.05) (Table 1).

**Table 1:** Demographic data of the patients

Variable	PL group	AL group	*P* value
Sex	Male, 9; female, 26	Male, 3; female, 26	0.12
Age (years)	67.1 ± 9.9	67.4 ± 10	0.79
Body mass index (kg/m^2^)	26.5 ± 5.2	22.6 ± 4.7	0.004
Diagnosis	OA, 30; ION, 4; RDC 1	OA, 18; ION, 4; RDC, 1; RA, 4	0.23
Preoperative leg discrepancy (mm)	− 9.9 ± 9.1	− 6.5 ± 7.2	0.02
Preoperative offset (mm)	46.9 ± 7.4	48.7 ± 8.7	0.23

*AL* anterolateral approach, *ION* idiopathic osteonecrosis of the hip, *OA* osteoarthritis, *PL* posterolateral approach, *RA* rheumatoid arthritis, *RDC* rapidly destructive coxarthrosis

The postoperative muscle volume in the PL group of the G-max (56.2 mm^2^/kg) was significantly higher than the preoperative muscle volume (45.6 mm^2^/kg) (Table 2). The postoperative HU of the internal obturator (13.7) and external obturator (13.5) in the PL group were significantly lower than the preoperative HU (internal obturator, 36.8; external obturator, 31) (Table 3). The postoperative muscle volume in the AL group of the G-max (55 mm^2^/kg), G-med (42.5 mm^2^/kg), and tensor fasciae latae (7.8 mm^2^/kg) significantly increased than the preoperative muscle volumes (G-max, 48.1mm^2^/kg; G-med, 40.5mm^2^/kg; tensor fasciae latae, 6.0 mm^2^/kg) (Table 2). The postoperative HU of G-med (33.7) and tensor fasciae latae (34.6) in the AL group were significantly higher than the preoperative HU (G-med, 29.2; tensor fasciae latae, 25.6). In contrast, the postoperative HU of the G-max (12.9) and external obturator muscle (23.8) significantly decreased compared to the preoperative values (G-max, 16.5; external obturator muscle, 29.2) (Table 3). The intraobserver and interobserver reliabilities were evaluated using ICC and were > 0.9 for each parameter, indicating an acceptable level of reliability [12]. The intraobserver and interobserver muscle volumes were 0.981 (95% CI, 0.977–0.996) and 0.967 (95% CI, 0.916–0.991), respectively. The intraobserver and interobserver HU values were 0.99 (95% CI, 0.982–0.996) and 0.918 (95% CI, 0.815–0.998), respectively.

**Table 2:** Comparison of muscle volume between preoperative CT and postoperative CT

CT evaluation of muscle volume (mm^2^/kg)	Preoperative muscle volume	Postoperative muscle volume	*P* value
**PL group**			
Gluteus maximus	45.6 ± 9.4	56.2 ± 11.3	0
Gluteus medius	37 ± 8	37.9 ± 8.5	0.334
Tensor fasciae latae	5.9 ± 1.8	6.4 ± 2	0.06
Internal obturator muscle	12.6 ± 3.4	9.7 ± 2.7	0
External obturator muscle	24.5 ± 7.6	23.4 ± 6.8	0.318
**AL group**			
Gluteus maximus	48.1 ± 6.7	55 ± 8.5	0
Gluteus medius	40.5 ± 9.5	42.5 ± 10	0.027
Tensor fasciae latae	6 ± 2.3	7.8 ± 2.3	0.003
Internal obturator muscle	14.7 ± 4.3	14.4 ± 4.3	0.304
External obturator muscle	26.8 ± 6.5	26.2 ± 27	0.787

*AL* anterolateral approach, *CT* computed tomography, *PL* posterolateral approach

**Table 3:** Comparison of HU between preoperative CT and postoperative CT

CT evaluation of HU	Preoperative HU	Preoperative HU	*P* value
**PL group**			
Gluteus maximus	14.5 ± 12.4	17 ± 12.3	0.17
Gluteus medius	24.6 ± 11.4	25.3 ± 11.1	0.79
Tensor fasciae latae	27.2 ± 16.4	23.3 ± 18.3	0.174
Internal obturator muscle	36.8 ± 10.8	13.7 ± 17.9	0
External obturator muscle	31 ± 11.5	13.5 ± 16.6	0
**AL group**			
Gluteus maximus	16.5 ± 17.5	12.9 ± 16.1	0.04
Gluteus medius	29.2 ± 12.8	33.7 ± 10.5	0.002
Tensor fasciae latae	25.6 ± 13.4	34.6 ± 24.2	0.048
Internal obturator muscle	34.3 ± 12.2	31.5 ± 14.8	0.24
External obturator muscle	29.2 ± 11.2	23.8 ± 16	0.04

*AL* anterolateral approach, *CT* computed tomography, *HU* Hounsfield units, *PL* posterolateral approach

## Discussion

In our study, the postoperative muscle volume of the G-max increased in the PL group. In contrast, the postoperative HU of the internal obturator and external obturator decreased. In the AL group, the postoperative muscle volume of the G-max, G-med, and tensor fasciae latae increased. Although the postoperative HU of the G-med and tensor fasciae latae in the AL group increased, the postoperative G-max and external obturator muscle decreased.

Some reports investigated pre- and postoperative muscle volumes using CT [5, 12]. Rasch et al. performed CT scans on patients 2 years after performing THA and reported that there were no significant increase in the G-max and G-med [12]. This could be because their sample size was small with only 20 participants. Not only that, the authors evaluated the muscles of the hip at different points compared to the points used in our study [12]. They evaluated the G-max and G-med at the middle between the ASIS and the greater trochanter [12]; this might explain their different results. There is a possibility that 2D CT cannot accurately evaluate the muscle volume. However, Ogawa et al. compared the 2D CT evaluation of muscular atrophy and fatty degeneration to the 3D evaluation [13] and reported that 64% of the muscles of the hip and femur exhibited a strong correlation between the 2D and 3D CT measurements of muscular atrophy, and 71% of the muscles of the hip and femur exhibited a strong correlation between the 2D and 3D CT measurements of muscular degeneration. Particularly, they reported that the strongest correlation point for the G-max was around the greater trochanter and that for the G-med was around the ASIS [13]. Therefore, in our study, these muscles were evaluated at the same point as reported in their study.

Uemura et al. performed CT scans on patients more than 2 years after performing THA and reported that postoperative muscle volumes of the G-max and G-med significantly increased than preoperative muscle volumes [5]. Our results were similar in that the postoperative muscle volumes of the G-max and G-med significantly increased. However, the participants underwent several surgical methods (THA and hip resurfacing arthroplasty) and several approaches (PL and direct anterior approach) [5]. In addition, the authors did not evaluate postoperative muscle degeneration. Therefore, whether surgical approaches affect postoperative muscle volume and degeneration remains unclear. Our results showed the PL approach to be more beneficial than the AL approach for the G-max because AL led to degeneration of the G-max. In contrast, the AL approach was more beneficial than the PL approach for the G-med and tensor fasciae latae because both of their muscle volume and HU were increased in the AL group. Not only that, the AL approach is more beneficial than the PL approach when regarding the internal obturator muscle because its postoperative muscle volume and HU were maintained when using the AL approach. On the other hand, the postoperative muscle volume and HU of the internal obturator muscle decreased when using the PL approach. This could be because the PL approach involves the resection of the short rotators; thus, the muscle volume and HU would have been decreased. Some authors reported that the AL approach may complicate the injury of the inferior branch of the superior gluteal nerve and cause abductor weakness [14–16]. However, the postoperative muscle volume and HU of the G-med and tensor fasciae latae increased. Thus, the AL approach did not significantly affect the decrease in the muscle volume and degeneration of the G-med.

There are some limitations to this study. First, this is a retrospective study and sample size was small. However, this study is the first report to evaluate muscle volume and degeneration after THA by using CT. Thus, we believe that this study will have a great impact on evaluation of muscles around the hip after THA. Second, this study includes some kind of diagnosis, which might affect muscle volume and degeneration. However, there were no significant differences in patients’ characteristics between the two groups. Third, leg discrepancy and lateral offset might have affected the results because they affect the tension of hip muscles. Fourth, surgeons performing AL and PL surgeries were not the same surgeons, which may have affected the results. However, the hip surgeons have sufficient years of experience (>100 cases). Additionally, both the surgeons participated in all the surgeries, and the surgical procedures were unified. Fifth, in this study, CT was performed only once after THA. Some studies reported that the muscle strength around the hip, walking speed, cadence, stride length, and hip range of motion reached the peak in 6 months after THA [17, 18]. Thus, we believe that regardless of the type of approach, the physical function of the hip recovered in 6 months after THA; therefore, the timing of CT used in this study was appropriate. However, once a muscle is resected, recovery of muscle volume and degeneration may take longer than recovery of physical function. Thus, it is preferable to evaluate the changes in muscle volume and degeneration by performing CT several times at regular intervals, such as 3, 6, and 12 months, after THA. We will be performing CT several times to reveal this transition in muscle volume and degeneration.

## Conclusion

In summary, surgical approaches to THA affect postoperative muscle volume and degeneration around the hip. Postoperative muscle volume and degeneration varied depending on the approach. The PL approach leads to degeneration of the internal and external obturator. The AL approach is more beneficial for recovering the G-med, tensor fasciae latae, and internal obturator muscle than the PL approach.

## Data Availability

All data generated or analyzed during this study are included in this published article.

## Abbreviations

AL: Anterolateral
APP: Anterior pelvic plane
ASIS: Anterior superior iliac spine
BMI: Body mass index
CI: Confidence interval
CSAs: Cross-sectional areas
CT: Computed tomography
G-max: Gluteus maximus
G-med: Gluteus medius
HU: Hounsfield units
ICCs: Intraclass correlation coefficients
PL: Posterolateral
THA: Total hip arthroplasty
